# SIRPα and PD1 expression on tumor-associated macrophage predict prognosis of intrahepatic cholangiocarcinoma

**DOI:** 10.1186/s12967-022-03342-6

**Published:** 2022-03-22

**Authors:** Hui Yang, Meimei Yan, Wei Li, Linping Xu

**Affiliations:** 1 Department of Gastroenterology, Zhengzhou University People’s Hospital and Henan Provincial People’s Hospital, Zhengzhou, 450003 Henan China; 2 Department of Research and Foreign Affairs, The Affiliated Cancer Hospital of Zhengzhou University and Henan Cancer Hospital, Zhengzhou, 450008 China; 3 Department of Hematology, The First Affiliated Hospital of Zhengzhou University, Zhengzhou, 450052 Henan China

**Keywords:** TAMs, Phagocytosis checkpoints, SIRPα, PD1, SIGLEC10

## Abstract

**Background:**

The phagocytosis checkpoints of CD47/SIRPα, PD1/PDL1, CD24/SIGLEC10, and MHC/LILRB1 have shown inhibited phagocytosis of macrophages in distinct tumors. However, phagocytosis checkpoints and their therapeutic significance remain largely unknown in intrahepatic cholangiocarcinoma (ICC) patients.

**Methods:**

We analyzed sequencing data from the Cancer Genome Atlas (TCGA) and identified differently expressed genes between tumors and para‐tumors. Then, we investigated the expression of CD68, SIRPα, PD1, and SIGLEC10 by IHC in 81 ICC patients, and the clinical significance of these markers with different risk factors was also measured.

**Results:**

Tumor infiltration immune cells analysis from the TCGA data revealed that macrophages significantly increased. Further analysis showed that M0 macrophages were significantly higher and M2 macrophages were significantly lower in ICC compared with paracancerous tissues, while there was no significant difference in M1 macrophages. We then examined some of M1 and M2 markers, and we found that M1 markers (iNOS, TNF, IL12A, and B) increased, while M2 markers (ARG1 and CD206) decreased in ICCs compared with paracancerous tissues. Furthermore, the expression of CD68, SIRPα, PD1, and SIGLEC10 increased significantly, but LILRB1 expression did not. We also examined the expression of CD68, SIRPα, PD1, and SIGLEC10 in 81 ICC patients by IHC, which revealed a similar expression pattern to that which emerged from the TCGA data. Upon analyzing the correlation between these markers and the progression of ICC patients, we found that the high expression of CD68, SIRPα, and PD1 are correlated with poor progression among ICC patients, while SIGLEC10 shows no correlation. More SIRPα^+^ or PD1^+^ TAMs were observed in the tumor tissues of ICC patients with HBV infections compared to non‐HBV‐infected patients. Multivariate analysis indicated that SIRPα and PD1 expression are independent indicators of ICC patient prognosis.

**Conclusion:**

Hyperactivated CD47/SIRPα and PD1/PD‐L1 signals in CD68^+^ TAMs in tumor tissues are negative prognostic markers for ICCs after resection. Furthermore, anti-CD47 in combination with anti-PD1 or CD47/PD1 bispecific antibody (BsAb) may represent promising treatments for ICC. Further studies are also required in the future to confirmed our findings.

## Introduction

Intrahepatic cholangiocarcinoma (ICC) is an aggressive and invasive malignant tumor occurring within the liver, and it represents the second most common primary malignancy of the liver [1]. In the last decade, ICC has become a major global concern due to its increasing rate of diagnosis and mortality [2]. Due to the insidious nature of the onset of ICC, most patients have already reached the advanced stage by the time of their diagnosis, and only a small proportion of patients’ tumors can be surgically removed [2, 3]. Moreover, the recurrence rate after surgical resection remains high, with only 5% of patients surviving for more than five years, and the prognosis remains very poor for patients with ICC who cannot be resected due to the lack of effective treatments [2, 3]. Importantly, prognostic indicators for post-operative ICC are still not fully understood.

The tumor microenvironment is complex and is regulated by multiple immune cells [4]. It plays an important role in supporting the growth of tumor cells during malignant tumorigenesis and progression [5]. Among the innate and adaptive immune cells recruited to the tumor’s interior, macrophages are particularly abundant and are present at all stages of tumor progression, and these are also known as tumor-associated macrophages (TAMs) [6, 7]. Clinical studies and experimental mouse models suggest that these macrophages often play a pro-tumoral role [8]. Several markers of TAMs such as CD206 [9], CD163 [10], ARG1 [11], and MARCO [12, 13] have been significantly correlated with aggressive tumor phenotypes and worse prognosis in tumors. In recent years, many studies have revealed that tumor cells evade phagocytosis by TAMs through high expression of phagocytosis checkpoints. Studies have indicated that phagocytosis checkpoints mainly include CD47/SIRPα axis [14], PD-1/PD-L1axis [15], CD24/SIGLEC10 axis [16], and MHC-I/LILRB1 axis [17]. Numerous studies have reported PD1/PD-L1signaling in CD3^+^ T cells and its therapeutic and prognostic significance [18, 19]. Additionally, the significant increase in CD47 expression in tumors has been detected, showing their positive correlation with poor disease progression [14, 20, 21]. However, the expression of phagocytosis checkpoints by TAMs and their correlation with ICC prognosis remains unclear.

In the present study, we first analyzed the various transcriptional expressions of ICC and paracancerous tissue published by previous studies available on the Cancer Genome Atlas (TCGA) databases. The cell type proportion were also examined. And then, we investigated the clinical relevance and prognostic significance of phagocytosis checkpoints expressed by CD68^+^TAMs in patients diagnosed with ICC. Our study highlights that the phagocytosis checkpoints of CD47/SIRPα and PD-1/PD-L1 axes are highly correlated with poor progression among ICC patients, while those of the CD24/SIGLEC10 and MHC-I/LILRB1 axes are not. Therefore, anti-CD47 in combination with anti-PD1 or CD47/PD1 bispecific antibodies (BsAbs) may present a novel and promising treatment option for ICC. Further studies are needed to confirm these findings.

## Materials and methods

### Differential expression gene analysis of ICC and paracancerous tissues from TCGA datasets

RNA-seq was analyzed as our previously described [22–24]. Briefly, the Limma package (version 3.40.2) of R software was used to study the differential expression of mRNAs [25]. The adjusted *P*-value was analyzed to correct for false positive results in TCGA or GTEx. “Adjusted *P* < 0.05 and Log2 (Fold Change) > 1 or Log2 (Fold Change) <  − 1” were defined as the thresholds for the screening of differential mRNA expression. To further confirm the underlying function of potential targets, the data was analyzed via functional enrichment. The Kyoto Encyclopedia of Genes and Genomes (KEGG) Enrichment Analysis is a practical resource for analytical study of gene functions and associated high-level genome functional information. To better understand the carcinogenesis of mRNA, the ClusterProfiler package (version: 3.18.0) in R was employed to analyze to enrich the KEGG pathway.

### Kaplan–Meier analysis of gene signature from TCGA datasets using the survival and survminer packages in R

Thirty-six ICCs from TCGA datasets were included in this study. Overall survival time (OS) was compared between the high and low TAM-related markers by Kaplan–Meier analysis as previously described [26, 27]. The software is survival and survminer packages in R. The median was selected as the cutoff value for high or low TAM-related markers.

### Immune infiltration estimations using CIBERSORT, QUANTISEQ, and MCPCOUTER

CIBERSORT, QUANTISEQ, and MCPCOUTER, which was used to analyze the immune cells infiltration in ICCs as previously described [28–31]. The immunedeconv, an R package was implemented by R foundation for statistical computing (2020) version 4.0.3 and software packages ggplot2 and pheatmap. And these methods were used in the present study.

### Patients and tumor samples

The sample consisted of 81 consecutive ICC patients who underwent curative resection from June 2, 2016 and December 30, 2019 at the affiliated cancer hospital of Zhengzhou University. This study was approved by the Ethics Committee at the affiliated cancer hospital of Zhengzhou University. All methods and procedures associated with this study were conducted in accordance with the Good Clinical Practice guidelines and accorded with the ethical principles of the Declaration of Helsinki as well as local laws. All enrolled patients were pathologically diagnosed with ICC and were not administered any anti-cancer treatments before surgery. After surgery, follow-ups were made on the patients to examine tumor status every 3 months for the first 2 years and every 6 months from year 3 to year 5. After 5 years, follow-ups were performed every year. The last follow-up took place on October 1, 2021.

### Immunohistochemistry and quantification of CD68, SIRPα, PD1 and SIGLEC10 density

Formalin-fixed and paraffin-embedded sections of intrahepatic cholangiocarcinoma tissue and paracancerous tissue (5 μm thick) were dewaxed and rehydrated. Antigen retrieval was performed by heating the slides in 10 mM Tris buffer with 1 mM EDTA (pH 9) in a streamer for 20 min. Inhibition of endogenous peroxidase activity was achieved by immersion in 3% H_2_O_2_ for 5 min. After washing with Tris-buffered saline (TBS) containing Tween, endogenous biotin was inhibited by sequential incubation with 0.1% anti-biotin protein and 0.01% biotin (Dako, Glostrup, Denmark), respectively, for 10 min at room temperature. Other non-specific binding sites were blocked with 3% skimmed milk powder for 30 min at room temperature. The tissue section of the intrahepatic cholangiocarcinoma tissue and paracancerous tissue were incubated with the monoclonal mouse antibody anti-human CD68 (Abcam, Clone# EPR20545, Cat# ab213363), SIRPα (Abcam, Clone# EPR22930-163, Cat# ab260039), PD1(Abcam, Clone# NAT105, Cat# ab52587), and SIGLEC10 (ThermoFisher, Cat# PA5-55,501) for one night at 4 ℃. Subsequently, the sections were serially rinsed and incubated with second antibodies. Immunohistochemical staining was evaluated independently by two experienced pathologists blinded to the patients’ clinical characteristics and outcomes. A histochemistry score (H-score) based on a combination of the percentage of positive stained cells and their staining intensity was calculated for the semiquantitative analysis as previously described [32–34]. H-Score(H-SCORE = ∑percentage[0–100%] × intensity (1–3)) = (percentage of weak intensity cells × 1) + (percentage of moderate intensity cells × 2) + (percentage of strong intensity cells × 3).The median H-score was selected as the cutoff value for high or low CD68, SIRPα, PD1, and SIGLEC10 expression.

### Follow-up and survival analysis of ICC patients

Follow-up and survival analysis of ICC Patients were consistent with previous studies [13, 35]. After surgery, the patients were checked regularly. In the first two years, follow-up evaluations were measured every 3 months. From 2 to 5 years after surgery, the follow-up tests of ICC patients were examined every 6 months. Beyond 5 years, the follow-up tests of ICC patients were measured every year. The follow-up tests included complete blood examinations, tumor biomarkers, and chest and abdominal computed tomography scans. If follow-up evaluations revealed metastatic disease and/or local recurrences, other therapies were applied, including conventional therapies (surgery, chemotherapy, and radiotherapy) as well as targeted and immunotherapy. Disease-free survival (DFS) was calculated from the date of surgery to the time of recurrence or metastasis, and patients who were alive and in a stable state were censored at the time of last contact [13, 35]. Overall survival (OS) was calculated using the date of surgery to the time of death, and patients who were alive at the time of last contact were censored [13, 35]. DFS and OS were calculated using the Kaplan–Meier analysis. The final follow-up was performed on October 1, 2021.

### Statistical analysis

GraphPad Prism 9.0 software (GraphPad Software, Inc.) and SPSS® 24.0 software were used to perform the statistical analysis. Quantification of CD68, SIRPα, PD1, and SIGLEC10 density were analyzed via *t*-test. DFS and OS were calculated using the Kaplan–Meier estimator. Univariable and multivariable Cox proportional hazards regression models were used to estimate hazard ratios along with associated confidence intervals and *p*-values. Student’s t-test and chi-square (χ2) test were employed for inferential statistical analysis. For all data, *P* < 0.05 was used to indicate a statistically significant difference.

## Results

### Identification of differentially expressed mRNAs and signal pathways of ICCs

**Fig. 1:**
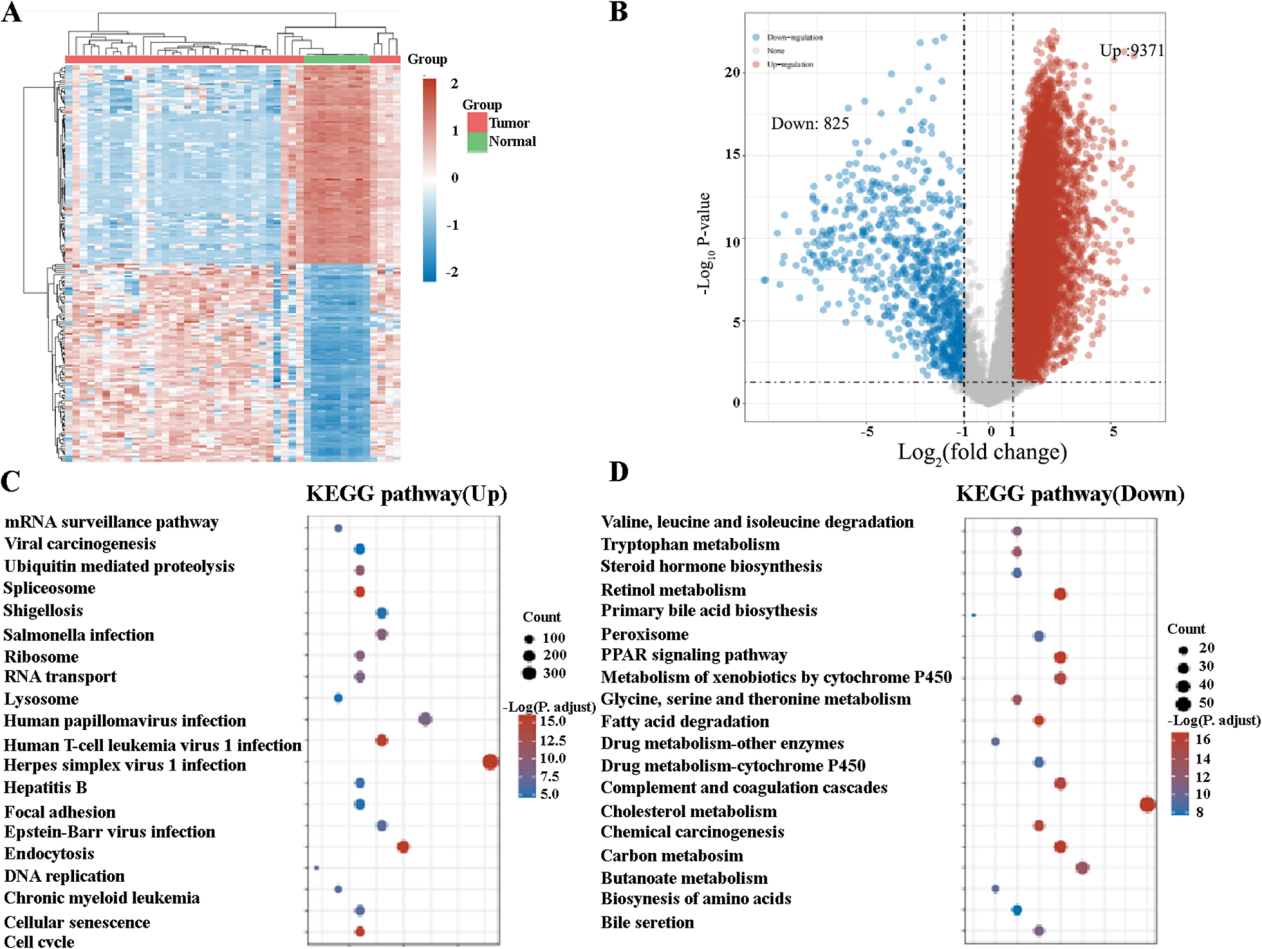
Differentially expressed genes and signal pathways between ICCs and adjacent tissues. **A** Heatmap demonstrating the differentially expressed genes between ICCs and adjacent tissues. **B** Volcanoes indicating the 9371 upregulated genes and 825 down-regulated genes in ICCs compared with adjacent tissues. **C** The top 20 upregulated pathways in ICCs. **D** The top 20 downregulated pathways in ICCs

To begin this study, we first retrieved the transcriptome profiling data of ICC patients from the Cancer Genome Atlas (TCGA) database. Nine normal and 36 tumor samples were included. Next, the data was analyzed using R software to study the differential expression of mRNAs. A total of 16,897 genes were identified, and 10,196 were distinguished as differentially expressed mRNAs (DEmRNAs) (Fig. 1A), of which 9,371 were upregulated and 825 were downregulated (Fig. 1B). Enriched KEGG signaling pathways were selected to demonstrate the primary biological actions of major potential mRNA. The upregulated pathways potentially related to TAMs include lysome and endocytosis (Fig. 1C). The downregulated pathways potentially related to TAMs include the PPAR signaling pathway (Fig. 1D). These results suggested that TAMs may play a significant role in the progression of ICCs.

### Analyses of cell type proportion, M1, M2 markers and phagocytosis checkpoints between normal and cancer tissues

**Fig. 2:**
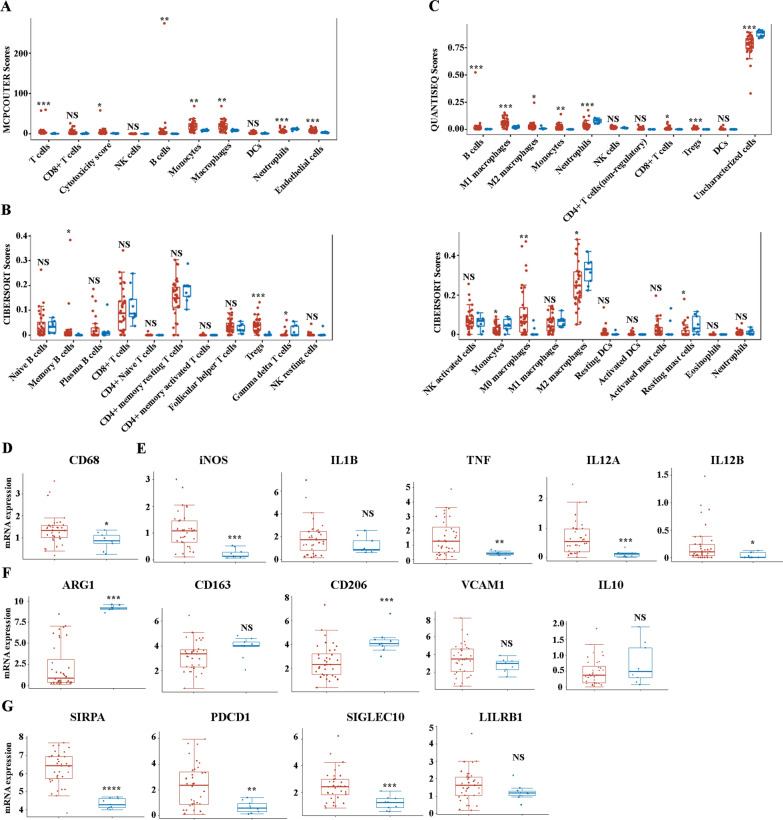
The selective M1, M2 and “don’t eat me” signal molecules expressed by TAMs in ICCs. **A** The characterization of the TIICs signature defined by MCPCOUTER score. **B** The characterization of the TIICs signature defined by CIBERSORT score. **C **The characterization of the TIICs signature defined by QUANTISEQ score. **D** The mRNA expression of total macrophage marker, CD68. **E** The mRNA expression of M1 markers, iNOS, IL1B, TNF, IL12A, and IL12B in ICCs. **F** The mRNA expression of M2 markers, ARG1, CD163, CD206, VCAM1, and IL10 in ICCs. **G** The mRNA expression of “don’t eat me” signal molecules, SIRPα, PDCD1, SIGLEC10, and LILRB1

Next, we analyzed the characterization of the tumor-infiltrating immune cells (TIICs) signature in ICCs using three methods, CIBERSORT, QUANTISEQ, and MCPCOUTER as previously described [28–31]. MCPCOUTER analysis demonstrated that macrophages/monocytes significantly increased in ICCs (Fig. 2A). CIBERSORT analysis showed increased infiltration of M0 macrophages in ICCs, and decreased infiltration of M2 macrophages in ICCs, while there was no significant difference in the infiltration of M1 macrophages (Fig. 2B). However, QUANTISEQ analysis indicated that both M1 and M2 macrophages significantly increased in ICCs (Fig. 2C). Then, we analyzed the expression of macrophage-related markers. CD68 is considered the gold standard marker of human total macrophages, and the expression of CD68 increases in ICC patients compared with normal controls, which is consistent with the TIICs analysis (Fig. 2D). We then analyzed the expression levels of M1 and M2 markers between normal and cancer tissues. Due to the increased scavenging capabilities of M2 macrophages, CD163, CD206, VCAM1, ARG1, IL-10, and CD204 have been proposed as markers of M2-type macrophages [36]. While M1 macrophages have been reported to have increased antigen processing, presentation, and killing properties. And IL-1β, iNOS, TNF, IL12A and B, CD80, CD83, and CD40 are considered typical markers of M1 macrophages [37]. When analyzing the expression levels of M1 markers between ICC and normal control, we found that iNOS, TNF, IL12A, and IL12B significantly increased, but IL-1β did not change (Fig. 2E). Furthermore, the M2 markers of CD163, IL10, and VCAM1 did not change between ICC patients and normal control. CD206 increased in ICC compared with normal control, but ARG1 decreased in ICC (Fig. 2F). Taken together, these findings suggest that M1 or M2 macrophages may not be a good biomarker to predict the progression of ICCs. Phagocytosis checkpoints, including PDCD1, LILRB1, SIGLEC10, and SIRPα are significantly for the function of TAMs in many cancers [14–17]. Finally, we examined the expression of phagocytosis checkpoints expressed by TAMs. Notably, of these four phagocytosis checkpoints, the expression of PDCD1, SIGLEC10, and SIRPα significantly increased in ICC compared with normal control. However, there was no significant difference in LILRB1 expression (Fig. 2G).

### Prognostic factors of differentially expressed M1 and M2 markers and phagocytosis checkpoints in cancer tissues

**Fig. 3:**
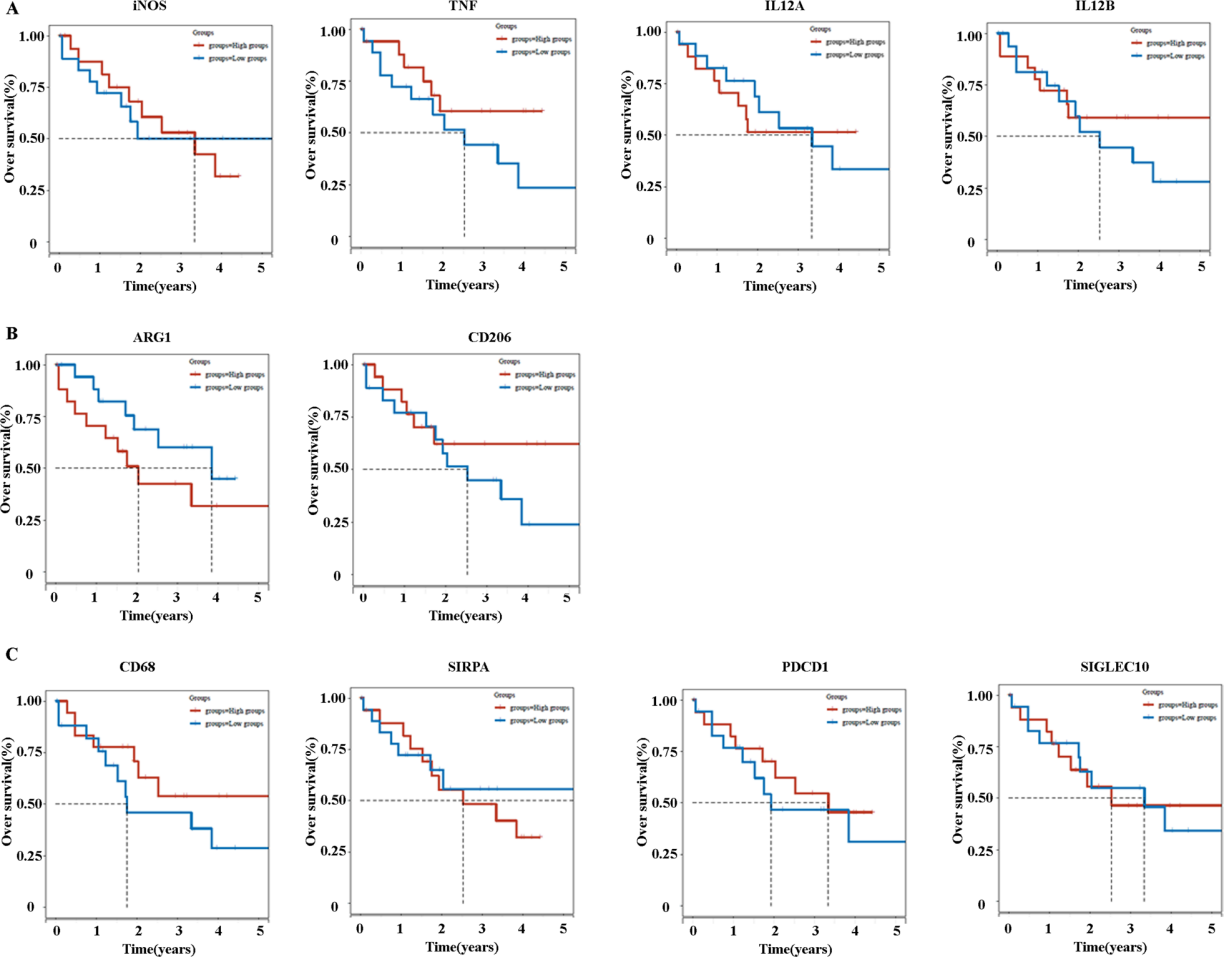
The overall survival time of ICCs based on high and low expression of TAM markers. **A** The OS of ICCs between high expression and low expression of iNOS, TNF, IL12A, and IL12B. **B** The OS of ICCs between high expression and low expression of ARG1 and CD206. **C** The OS of ICCs between high expression and low expression of CD68, SIRPα, PDCD1, and SIGLEC10

We examined the prognostic impact of differentially expressed M1, M2 markers and phagocytosis checkpoints in cancer tissues in ICC patients from the Cancer Genome Atlas (TCGA) database. The Kaplan–Meier survival analysis and log-rank test were used to compare the survival rates between high expression (N = 18) and low expression (N = 18) groups of ICC patients. Interestingly, the Kaplan–Meier analysis indicated that patients exhibited a similar survival time based on the expression levels of iNOS, TNF, IL12A, IL12B, ARG1, CD206, CD68, SIRPα, PDCD1, and SIGLEC10 (Fig. 3A–C).

### Patient characteristics of ICC from the affiliated cancer hospital of Zhengzhou university

Given that the TCGA database had limited data on ICC patients, we sought to assess the role of “don’t eat me” molecules by using data from additional patients in China. A total of 81 patients between June 2, 2016 and December 30, 2019 were used in this study, including 47 (58.02%) males and 34 (41.98%) females. The median age was 61 years. All the patients received surgery. Prior to surgery, 20 (24.69%) patients had HBV infections, 10 (12.35%) patients had cirrhosis, and two patients had a history of hepatitis C infections. A total of 62 (78.48%) patients had elevated serum CA19-9, seven patients had elevated serum CEA (8.64%), and 55 (67.9%) patients had elevated serum CA724. Additionally, 69 patients had elevated serum total bilirubin, 72 patients had elevated serum direct bilirubin, and 49 patients had elevated serum indirect bilirubin. Moreover, 56 patients had elevated serum alanine transaminase (ALT), and 50 patients had elevated serum aspartate transaminase (AST). Finally, 14 patients were classified as Child–Pugh grade A, and 67 patients were Child–Pugh grade B.

Regarding tumors, all patients had a solitary tumor, and seven patients had larger tumors (> 5 cm). A total of 54 patients were TNM stage I or II, while 27 patients were TNM stage III. In addition, 54.32% of tumors had high to intermediate histopathological grading, and 45.68% of tumors had intermediate to low histopathological grading. In addition, 19.75% of patients had lymph node invasion, and 20.99% of patients had microvascular invasion. Patients’ detailed clinicopathologic characteristics are showed in Table 1.

**Table 1:** Patient characteristics

Characteristics	No. of patients	%
Gender		
Male	47	58.02
Female	34	41.98
Age (years)		
Median	61	
Range	44 ~ 82	
ECOG PS		
0	37	45.68
1	44	54.32
Size of primary tumor(cm)		
≥ 5	7	8.64
< 5	74	91.36
Histopathological grading		
High	6	7.41
Intermediate-high	5	6.17
Intermediate	33	40.74
Intermediate-low	24	29.63
Low	13	16.05
TNM stage		
I	45	55.56
II	9	11.11
III	27	33.33
Lymph node invasion		
Yes	16	19.75
No	65	80.25
Microvascular invasion		
Yes	17	20.99
No	64	79.01
HBV infection		
Yes	20	24.69
No	61	75.31
HCV infection		
Yes	2	2.47
No	79	97.53
Liver cirrhosis		
Yes	10	12.35
No	71	87.65
Child–Pugh grade		
A	14	17.28
B	67	82.72
CEA (ng/mL)		
≥ 10	7	8.64
< 10	74	91.36
CA199(U/mL)		
≥ 60	62	76.54
< 60	19	23.46
CA724(U/mL)		
≥ 14	55	67.90
< 14	26	32.10
ALT(U/L)		
≥ 80	56	69.14
< 80	25	30.86
AST(U/L)		
≥ 70	50	61.73
< 70	31	38.27
Total bilirubin(umol/L)		
≥ 42	69	85.19
< 42	12	14.81
Direct bilirubin(umol/L)		
≥ 16	72	88.89
< 16	9	11.11
Indirect bilirubin(umol/L)		
≥ 30	49	60.49
< 30	32	39.51

### Highly and differentially expressed phagocytosis checkpoints in ICCs

**Fig. 4:**
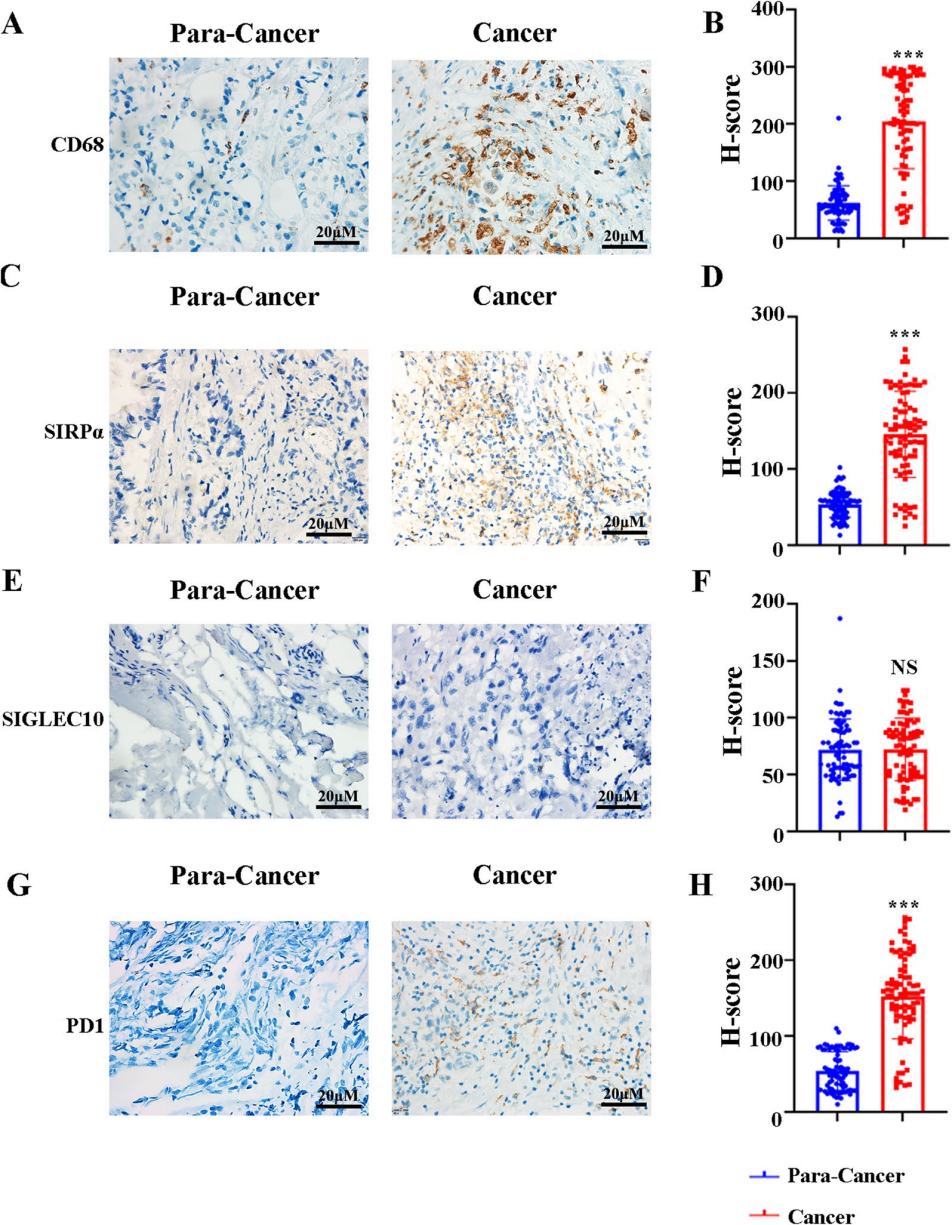
The expression of CD68, SIRPα, SIGLEC10, and PD1 on TAMs in ICCs as examined by IHC. **A** Representative IHC images of CD68 in ICCs and paracancerous tissues. **B** Quantitative analysis of H-score indicating the expression of CD68 in ICCs and para-cancer. **C** Representative IHC images of SIRPα in ICCs and para-cancer. **D** Quantitative analysis of H-score indicating the expression of SIRPα between ICCs and para-cancer. **E** Representative IHC images of SIGLEC10 in ICCs and para-cancer. **F** Quantitative analysis of H-score indicating the expression of SIGLEC10 between ICCs and para-cancer. **G** Representative IHC images of PD1 in ICCs and para-cancer. **H** Quantitative analysis of H-score indicating the expression of PD1 between ICCs and para-cancer

To further confirm the expression pattern of SIRPα, PD1, and SIGLEC10 in TAMs in ICCs, 81 ICC tissues and 81 paracancerous tissues were obtained from our hospital. First, H&E staining was performed to prove that the tissues taken were indeed paracancerous and cancerous. Then, immunohistochemistry (IHC) was performed with anti-CD68, anti-SIRPα, anti-SIGLEC10, and anti-PD1 antibodies to detect the phagocytosis checkpoints expression pattern in ICCs. IHC images of representative CD68, SIRPα, SIGLEC10, and PD1 from cancerous and paraneoplastic tissues are shown in Fig. 4A, C, E and G, receptively. H-score was used to semi-quantify the expression of CD68, SIRPα, SIGLEC10, and PD1. Quantitative analysis indicated that the H-scores of CD68, SIRPα, and PD1 in cancerous tissues significantly increased compared with paraneoplastic tissues (Fig. 4B, D, F, H), but this was not the case for SIGLEC10. These results reveal that ICC patients may highly express SIRPα and PD1 on TAMs in cancerous tissues.

### Relationship between phagocytosis checkpoints and clinicopathologic features of ICCs

The expression levels of CD68, SIRPα, and PD1 are diverse in each sample of ICCs. The median H-score was selected as the cutoff value for high or low expression of CD68, SIRPα, and PD1. Based on the cut-off value of each molecule, we divided the ICCs into high expression (CD68^high^, SIRPα^high^, and PD1^high^) and low expression subgroups (CD68^low^, SIRPα^low^, and PD1^low^). It is worth noting that high CD68 expression as well as high SIRPα were positively correlated with high TNM stage, lymph node invasion, high Child–Pugh stage, microvascular invasion, and HBV infection (Tables 2, 3). Consistent with previous studies, high PD1 expression was positively correlated with high TNM stage, lymph node invasion, and HBV infection (Table 4).

**Table 2:** Correlation of CD68 expression by TAMs with clinicopathological characteristics of ICCs

Characteristics (No. of patients)	CD68 ^hi^	CD68 ^low^	*P*-value
Gender			
Male	30	17	
Female	20	14	0.647
Age(years)			
≥ 60	20	18	
< 60	30	13	0.113
ECOG PS			
0	23	14	
1	27	17	0.941
Size of primary tumor (cm)			
≥ 5	3	4	
< 5	47	27	0.282
Histopathological grading			
Low	24	13	
Intermediate to high	26	18	0.594
TNM stage			
I + II	26	28	
III	24	3	0
Lymph node invasion			
Yes	14	2	
No	36	29	0.018
Microvascular invasion			
Yes	16	1	
No	34	30	0.002
HBV			
Yes	18	2	
No	32	29	0.003
HCV			
Yes	1	1	
No	49	30	0.73
Liver cirrhosis			
Yes	4	6	
No	46	25	0.131
Child–Pugh grade			
A	4	10	
B	46	21	0.005
CEA (ng/mL)			
≥ 10	4	3	
< 10	46	28	0.794
CA199(U/mL)			
≥ 60	36	26	
< 60	14	5	0.138
CA724(U/mL)			
≥ 14	35	20	
< 14	15	11	0.607
ALT(U/L)			
≥ 80	37	19	
< 80	13	12	0.229
AST(U/L)			
≥ 70	30	20	
< 70	20	11	0.684
Total bilirubin (umol/L)			
≥ 42	44	25	
< 42	6	6	0.365
Direct bilirubin (umol/L)			
≥ 16	46	26	
< 16	4	5	0.258
Indirect bilirubin (umol/L)			
≥ 30	33	16	
< 30	17	15	0.198

**Table 3:** Correlation of SIRPα expression by TAMs with clinicopathological characteristics of ICCs

Characteristics (No. of patients)	SIRPα^hi^	SIRPα^low^	*P*-value
Gender			
Male	26	21	
Female	16	18	0.463
Age (years)			
≥ 60	17	21	
< 60	25	18	0.228
ECOG PS			
0	19	18	
1	23	21	0.934
Size of primary tumor(cm)			
≥ 5	3	4	
< 5	39	35	0.618
Histopathological grading			
Low	20	17	
Intermediate to high	22	22	0.716
TNM stage			
I + II	22	32	
III	20	7	0.005
Lymph node invasion			
Yes	14	2	
No	28	37	0.001
Microvascular invasion			
Yes	15	2	
No	27	37	0.001
HBV			
Yes	17	3	
No	25	36	0.001
HCV			
Yes	1	1	
No	41	38	0.958
Liver cirrhosis			
Yes	4	6	
No	38	33	0.423
Child–Pugh grade			
A	2	12	
B	40	27	0.002
CEA (ng/mL)			
≥ 10	4	3	
< 10	38	36	0.769
CA199(U/mL)			
≥ 60	30	32	
< 60	12	7	0.26
CA724(U/mL)			
≥ 14	30	25	
< 14	12	14	0.48
ALT(U/L)			
≥ 80	31	25	
< 80	11	14	0.345
AST(U/L)			
≥ 70	26	24	
< 70	16	15	0.75
Total bilirubin (umol/L)			
≥ 42	36	33	
< 42	6	6	0.889
Direct bilirubin (umol/L)			
≥ 16	38	34	
< 16	4	5	0.637
Indirect bilirubin (umol/L)			
≥ 30	28	21	
< 30	14	18	0.238

**Table 4:** Correlation of PD1 expression by TAMs with clinicopathological characteristics of ICCs

Characteristics (No. of patients)	PD1^hi^	PD1^low^	*P*-value
Gender			
Male	28	19	
Female	18	16	0.552
Age(years)			
≥ 60	20	18	
< 60	26	17	0.478
ECOG PS			
0	23	14	
1	23	21	0.371
Size of primary tumor (cm)			
≥ 5	4	3	
< 5	42	32	0.984
Histopathological grading			
Low	20	17	
Intermediate to high	26	18	0.649
TNM stage			
I + II	24	30	
III	22	5	0.002
Lymph node invasion			
Yes	13	3	
No	33	32	0.027
Microvascular invasion			
Yes	11	6	
No	35	29	0.459
HBV			
Yes	18	2	
No	28	33	0.001
HCV			
Yes	1	1	
No	45	34	0.844
Liver cirrhosis			
Yes	6	4	
No	40	31	0.827
Child–Pugh grade			
A	6	8	
B	40	27	0.247
CEA (ng/mL)			
≥ 10	5	2	
< 10	41	33	0.413
CA199(U/mL)			
≥ 60	32	30	
< 60	14	5	0.089
CA724(U/mL)			
≥ 14	34	21	
< 14	12	14	0.184
ALT(U/L)			
≥ 80	33	23	
< 80	13	12	0.561
AST(U/L)			
≥ 70	30	20	
< 70	16	15	0.459
Total bilirubin (umol/L)			
≥ 42	38	31	
< 42	8	4	0.454
Direct bilirubin (umol/L)			
≥ 16	42	30	
< 16	4	5	0.428
Indirect bilirubin (umol/L)			
≥ 30	30	19	
< 30	16	16	0.319

### Prognostic implications of phagocytosis checkpoints in ICC patients

**Fig. 5:**
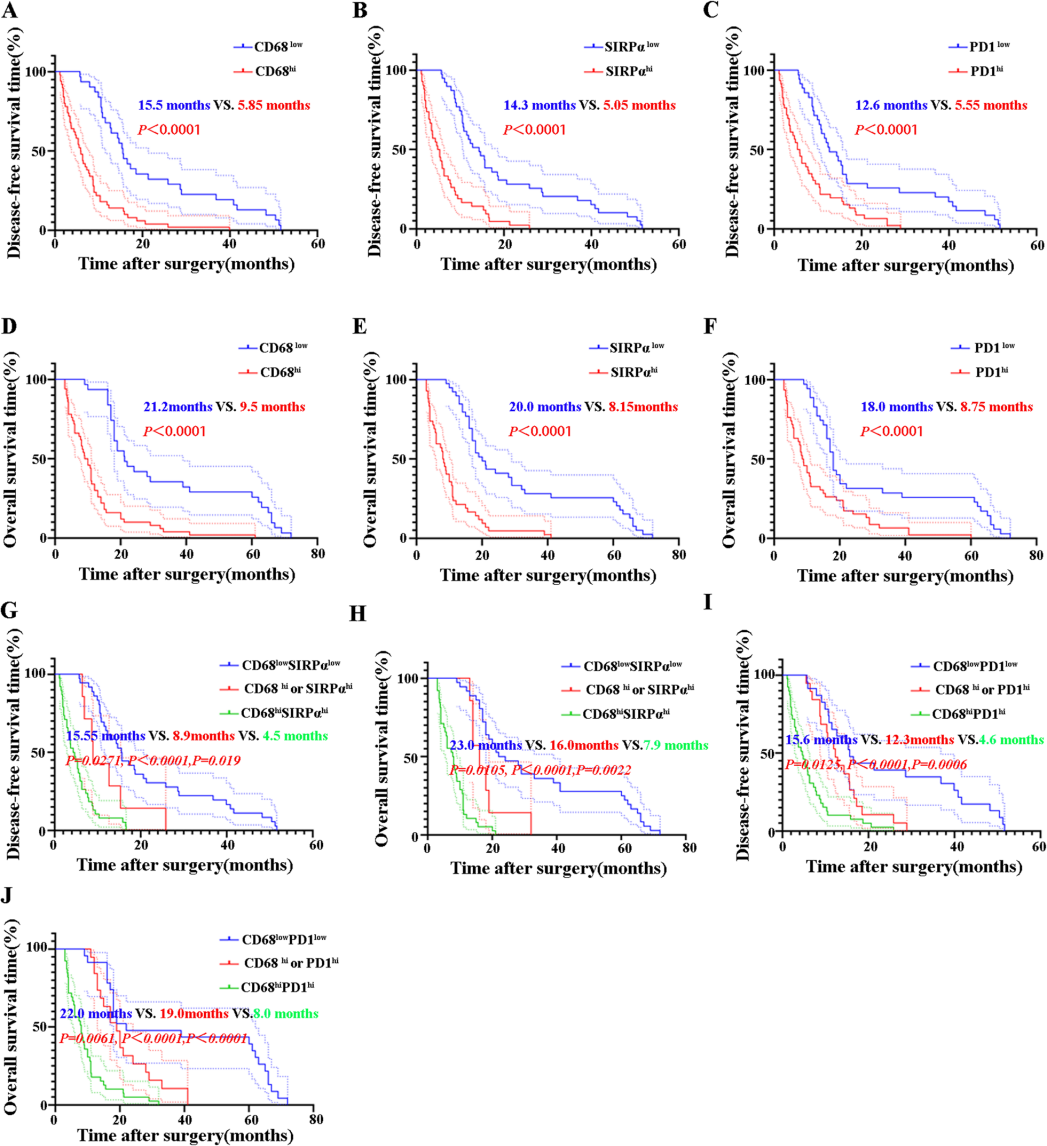
Survival analysis of ICCs based on high and low expression of CD68, SIRPα, and PD1. The median H-score was selected as the cutoff value for high or low expression of CD68, SIRPα, and PD1. **A** The DFS of ICCs between CD68^hi^ and CD68^low^. **B** The DFS of ICCs between SIRPα^hi^ and SIRPα^low^. **C** The DFS of ICCs between PD1^hi^ and PD1^low^. **D** The OS of ICCs between CD68^hi^ and CD68^low^. **E** The OS of ICCs between SIRPα^hi^ and SIRPα^low^. **F** The OS of ICCs between PD1^hi^ and PD1^low^. **G** The DFS of ICCs among CD68^hi^ SIRPα^hi^, CD68^hi^ or SIRPα^hi^ and CD68^low^ SIRPα^low^. **H** The OS of ICCs among CD68^hi^ SIRPα^hi^, CD68^hi^ or SIRPα^hi^ and CD68^low^ SIRPα^low^. **I** The DFS of ICCs among CD68^hi^ PD1^hi^, CD68^hi^ or PD1^hi^ and CD68^low^ PD1^low^. **J** The OS of ICCs among CD68^hi^ PD1^hi^, CD68^hi^ or PD1^hi^ and CD68^low^ PD1^low^

The final follow-up occurred on October 1, 2021. Up to October 1, 2021, all the patients died. The 1-year and 5-year OS rates among the 81 patients were 51.85% (42/81) and 12.35% (10/81), respectively. The 1-year and 5-year cumulative recurrence rates were 44.44% (36/81) and 100% (81/81), respectively. CD68^high^, PD1^high^, and SIRPα^high^ ICCs had shorter DFS (Fig. 5A–C) and OS (Fig. 5D–F) than CD68^low^, PD1^low^, and SIRPα^low^ patients (*P* < 0.05). The significance of PD1 expression in T cells among ICC patients has been reported in previous studies [38].

We also analyzed the prognostic roles of high expression of both CD68 and PD1 in ICC patients. Interestingly, ICC patients with high expression of both CD68 and PD1 showed a poorer prognosis compared to patients with high expression of only CD68 or PD1 and patients with low expression of both CD68 and PD1 (Fig. 5G,  P < 0.05). Similarly, ICC patients with high expression of both CD68 and SIRPα showed a poorer prognosis than patients with high expression of only CD68 or SIRPα and patents with low expression of both CD68 and SIRPα (Fig. 5H,  P < 0.05).

Univariate analysis showed that high TNM stage, lymph node invasion, high Child–Pugh stage, microvascular invasion, and HBV infection were risk factors for DFS and OS (Table 5). Notably, CD68, PD1, and SIRPα in ICCs were also correlated with DFS and OS (Table 5).

**Table 5:** Univariate analysis

Parameters	Hazard ratio	DFS		Hazard ratio	OS	
		95% CI	*P*-value		95% CI	*P*-value
TNM stage (I + IIVS.III)	1.65	(1.51, 2.16)	0.678	1.81	(1.32–2.45)	0.505
Lymph node invasion (yes vs no)	2.46	(1.29, 4.78)	< 0.001	2.86	(1.84–5.13)	< 0.001
Microvascular invasion (yes vs no)	1.87	(1.31–1.96)	0.728	1.63	(1.35–1.88)	0.285
HBV (yes vs no)	2.13	(1.45–4.23)	< 0.001	1.91	(1.63–4.15)	< 0.001
Child–Pugh grade (A vs. B)	1.84	(1.47–1.91)	0.123	1.21	(0.99–1.64)	0.415
CD68 (high VS low)	2.95	(1.87–4.65)	< 0.001	3.08	(1.95–4.87)	< 0.001
SIRPα (high VS low)	3.06	(1.86–5.02)	< 0.001	3.23	(1.96–5.35)	< 0.001
PD1 (high VS low)	2.39	(1.52–3.78)	< 0.001	2.36	(1.50–3.72)	< 0.001
CD68/SIRPα (double high VS others)	2.38	(0.77–7.36)	0.0251	5.05	(1.46–17.45)	0.0203
CD68/PD1 (double high VS others)	2.02	(1.04–3.94)	0.0682	2.12	(1.08–4.15)	0.0495

These risk factors from the univariate analysis were adopted as covariates in a multivariate Cox proportional hazards model. High TNM stage, lymph node invasion, high Child–Pugh stage, microvascular invasion, HBV infection, CD68, PD1, and SIRPα were independent prognostic indicators for DFS and OS. Along with combined expression of CD68/PD1 and CD68/SIRPα, the hyperactivated phagocytosis checkpoints was also an independent prognostic predictor for both DFS and OS (Table 6).

**Table 6:** Multivariate analysis

Parameters	Hazard ratio	DFS		Hazard ratio	OS	
		95% CI	*P*-value		95% CI	*P*-value
Lymph node invasion (yes vs no)	1.17	(0.824, 1.427)	0.612	1.54	(1.16–1.74)	0.308
HBV (yes vs no)	0.89	(0.45–1.23)	0.358	0.91	(0.63–1.15)	0.546
CD68 (high vs low)	1.58	(0.96–2.89)	0.002	2.11	(1.38–3.65)	0.001
SIRPα (high vs low)	1.78	(1.37–4.02)	0.003	1.55	(0.98–3.54)	0.004
PD1 (high vs low)	1.39	(1.35–2.66)	0.006	1.49	(1.14–2.68)	0.006
CD68/SIRPα (double high vs others)	1.28	(0.81–6.25)	0.016	2.16	(1.15–4.85)	0.018
CD68/PD1 (double high vs others)	–	–	–	1.89	(1.18–3.66)	0.034

## Discussion

Macrophages are the most plastic cell type in the body and are stimulated by their surroundings to polarize towards M1 or M2 [24, 39–41]. The ability to express distinct functional programs in response to different micro-environmental signals is a biological feature of macrophages, and it is typically manifested in pathological conditions such as infections and cancer [42]. The tumor microenvironment is often accompanied by inflammatory conditions [43]. Tumors induce alternatively activated M2 macrophages, but inflammatory conditions induce the classically activated M1 macrophage [44]. M1 macrophages are characterized by a high capacity to present antigens; high IL-12, IL-1β, and TNF production; and high expression of iNOS [42]. In contrast, M2 macrophages have poor antigen presenting capacity; have an IL-12^low^, IL-10^high^ phenotype; suppress inflammatory responses and Th1 adaptive immunity; actively scavenge among debris; and promote wound healing, angiogenesis, and tissue remodelling [42]. Furthermore, M2 macrophages highly express CD163, CD206, VCAM1, and ARG1. Therefore, this rigid distinction between M1 and M2 macrophages does not fully represent the continuum of functional states that macrophages can express and is a simplified view of these two extremes of polarization. Single cell analysis of pancreatic cancer patients demonstrated that the traditional M1 vs. M2 classification of macrophages might not appropriately reflect macrophages’ diversity [45]. In this study, we found that M1 markers such as iNOS, TNF, IL12A, and IL12B significantly increase in ICC compared with normal control. However, the M2 markers ARG1 and CD206 decrease in ICC. This data suggests that M1 and M2 markers may not be suitable progress markers for ICC patients.

Previous studies have confirmed that PD1/PD-L1 signals are hyper-activated in the tumor tissues of a large cohort of ICCs [38] and that ICC patients expressing high levels of PD1/PD-L1 signals have the poorest prognosis [38]. In addition, studies have determined that PD1^+^ T cells are enriched in ICC patients with HBV infections [38]. During a chronic HBV infection, these PD1^+^ T cells become exhausted, which likely results in decreased tumor responses during anti-PD1 immunotherapy [38]. Indeed, clinical studies have also demonstrated that only a minority of ICC patients show responses to the PD1 inhibitor pembrolizumab [46]. However, anti-viral therapies effectively prolong the OS of HBV-infected ICC patients [47]. These studies indicate that PD1^+^ T cells in ICC patients with HBV infections have likely lost their T cell function and may act as a marker for assaying the tumor response of PD1 inhibitors. In the present study, we found that CD68 and PD1 increase in ICC patients and that high expression of both CD68 and PD1 related to the poorest prognosis. *Gordon* et al. reported that PD-1 expression by TAMs inhibits phagocytosis and tumor immunity [15]. We also found that macrophages are significantly increased in ICCs and increased expression of CD68 and PD1 are correlated with HBV infection. Chronic HBV infection induces inflammatory conditions in the tumor microenvironment, which possibly leads to macrophage enrichment [48]. These macrophages with high PD1 expression induced by tumor cells were significantly inhibited in their phagocytosis [15]. Thus, our data suggests that CD68^+^PD1^+^ TAMs may also contribute to ICC progression via the inhibition of phagocytosis. Since our study only used single-plex IHC to analyze the expression of CD68 and PD1, further studies are also required to confirm our findings using multiplex assay in the future.

Although macrophages, granulocytes, dendritic cells, and monocytes can all express SIPRα, it is predominantly expressed on macrophages in tumors [49]. Furthermore, the function of the CD47/SIRPα axis was established in the late 2000s and has been termed the first tumor phagocytosis-related checkpoint (also known as the macrophage “don’t eat me” signal) [50]. A significant increase in CD47 expression has been detected in various hematological malignancies and solid tumors [20, 21]. In addition, CD47 overexpression is often correlated with poor clinical outcomes [20, 21]. CD47 was also highly expressed in cholangiocarcinoma patients [51]. The effectiveness of CD47-SIRPα blockage in macrophage-mediated cholangiocarcinoma removal was also proven in vitro and in vivo. [51] Significantly, anti-CD47-promoted phagocytosis was independent of macrophage subtype and could overcome TAM-promoting cancer effects, suggesting that SIRPα can be expressed by all macrophage subgroups [51]. Many studies have also indicated that macrophage deletion significantly inhibits CD47-mediated tumor remission [52] and that anti-CD47 therapy depends on the presence of macrophages. Although the significance of CD47 expression in tumors has been identified in many tumor types [14, 21, 51, 53, 54], the role of SIRPα in ICC patients remains unclear. In the present study, our data demonstrates that CD68 and SIRPα were more highly expressed in ICC patients compared with para-cancer controls. Furthermore, ICC patients simultaneously expressing high levels of CD68 and SIRPα had the poorest prognosis among all patients. These results suggest that CD68 and SIRPα may be poor progress markers, and the targeting macrophage phagocytosis checkpoint may be a promising treatment for ICC. However, monotherapy with anti-CD47 or SIRPα did not show significant anti-tumor activity in clinical studies [55, 56]. These findings can be explained as follows: (1) other phagocytosis checkpoints also play significant roles in ICC; (2) highly complex tumor immune microenvironment in ICC inhibit anti-tumor activity; (3) the intra-tumor mechanisms of ICC affect the sensitivity to anti-tumor drugs.

Although monotherapy with anti-CD47 or SIRPα has failed in clinical studies, anti-CD47 or SIRPα in combination with conventional therapies has shown significantly increased anti-tumor activities, especially in hematological malignancies [57–59]. Given that increased PD1 and SIRPα were observed in ICC patients in the present study, we speculate that anti-CD47 in combination with anti-PD1 may achieve better anti-tumor effects in ICC patients. Pre-clinical studies have confirmed that anti-CD47 enhances the anti-tumor efficacy of anti-PD1/PD-L1, including in melanoma, colon carcinoma, lung cancer, and high-Ep-CAM cancer cells [60–62]. Additionally, IBI322, a CD47/PD-L1 BsAb which attenuates CD47 activity in monovalent binding and blocks PD-L1 activity in bivalent binding, was designed by Wang et al. to enhance anti-tumor activity in PD-L1-expressing solid tumors both in vitro and in vivo [63]. Currently, IBI322 is in a Phase 1 dose escalation trial (NCT04328831). Together, these existing studies have confirmed that a combined anti-CD47/SIRPα and anti-PD1/PDL1 strategy enhances the anti-tumor activity among some solid tumors. Further preclinical studies and clinical studies are expected to fully validate the safety and effectiveness of this strategy both in ICC tumor models and human patients.

The significance of our research is that it provides both biomarkers to predict the prognosis of ICC patients and a new immunotherapeutic strategy for ICC. However, our study has certain limitations to consider. (1) Our results need to be validated in a randomized, controlled study using multiplex IHC assay. (2) The clinical efficacy of anti-CD47 in combination with anti-PD1 needs to be identified. (3) The complex immune profiles of ICC need to be fully uncovered.

In conclusion, our study indicates that the phagocytosis checkpoints of the PD1/PDL1 axis and CD47/SIRPα are enhanced in the tumor tissues of ICC patients and especially in HBV infection patients. High expressions of PD1 or SIRPα in ICC patients predicts poor progression. Furthermore, anti-CD47 in combination with anti-PD1 may be a novel and promising treatment for ICC. Although correlation among the expression of CD68, SIRPα, and PD1 was revealed, further studies evaluating the other SIRPα-expressing or PD1-expressing cells will improve the understanding of the association between its expression and the tumor microenvironment in ICCs. Further studies are needed to confirm these findings.

## Data Availability

All data generated in the study is included in the present article.

## Abbreviations

MHC-I: Major histocompatibility complex-I
OS: Overall survival time
PD1: Programmed cell death protein 1
PD-L1: Programmed death-ligand 1
SIRPα: Signal regulatory protein α
TAM: Tumor associated macrophage
